# Supplementary material for: Multi‐dataset identification of innovative feature genes and molecular mechanisms in keratoconus
Source: J Cell Mol Med. 2024 Sep 19;28(18):e70079. doi: 10.1111/jcmm.70079 (PMC11412914; doi:10.1111/jcmm.70079)
Supplement: Supplementary file 2 — Material S2. [file JCMM-28-e70079-s002.zip › 7.GSEA analysis/4.MYRF/KEGG_OLFACTORY_TRANSDUCTION.html]

Details for gene set KEGG\_OLFACTORY\_TRANSDUCTION[GSEA]

|  || Dataset | MYRF.group.cls#h\_versus\_l |
| Phenotype | group.cls#h\_versus\_l |
| Upregulated in class | h |
| GeneSet | KEGG\_OLFACTORY\_TRANSDUCTION |
| Enrichment Score (ES) | 0.23230158 |
| Normalized Enrichment Score (NES) | 0.7720468 |
| Nominal p-value | 0.9958159 |
| FDR q-value | 1.0 |
| FWER p-Value | 1.0 |
Table: GSEA Results Summary

  

Fig 1: Enrichment plot: KEGG\_OLFACTORY\_TRANSDUCTION      
 Profile of the Running ES Score & Positions of GeneSet Members on the Rank Ordered List

  

| SYMBOL | TITLE | RANK IN GENE LIST | RANK METRIC SCORE | RUNNING ES | CORE ENRICHMENT || 1 | OR52E8 | na | 270 | 0.666 | 0.0012 | Yes |
| 2 | CAMK2B | na | 283 | 0.659 | 0.0098 | Yes |
| 3 | OR10A3 | na | 315 | 0.646 | 0.0177 | Yes |
| 4 | OR51E1 | na | 726 | 0.512 | 0.0127 | Yes |
| 5 | OR52B2 | na | 740 | 0.508 | 0.0192 | Yes |
| 6 | OR5P2 | na | 750 | 0.506 | 0.0258 | Yes |
| 7 | OR8J1 | na | 1008 | 0.459 | 0.0246 | Yes |
| 8 | OR4N4 | na | 1031 | 0.455 | 0.0301 | Yes |
| 9 | OR2G2 | na | 1089 | 0.447 | 0.0345 | Yes |
| 10 | OR13F1 | na | 1095 | 0.446 | 0.0404 | Yes |
| 11 | OR52H1 | na | 1100 | 0.446 | 0.0464 | Yes |
| 12 | OR4F6 | na | 1259 | 0.425 | 0.0475 | Yes |
| 13 | OR1L8 | na | 1323 | 0.418 | 0.0514 | Yes |
| 14 | OR4K17 | na | 1341 | 0.415 | 0.0565 | Yes |
| 15 | OR10G3 | na | 1432 | 0.407 | 0.0594 | Yes |
| 16 | OR1D5 | na | 1448 | 0.405 | 0.0645 | Yes |
| 17 | OR56A5 | na | 1449 | 0.405 | 0.0700 | Yes |
| 18 | OR10A4 | na | 1672 | 0.385 | 0.0687 | Yes |
| 19 | OR6X1 | na | 1707 | 0.382 | 0.0729 | Yes |
| 20 | OR10AD1 | na | 1804 | 0.374 | 0.0752 | Yes |
| 21 | OR8D1 | na | 1816 | 0.372 | 0.0800 | Yes |
| 22 | OR9I1 | na | 1886 | 0.366 | 0.0829 | Yes |
| 23 | OR4K15 | na | 1903 | 0.365 | 0.0874 | Yes |
| 24 | OR6N2 | na | 1905 | 0.365 | 0.0923 | Yes |
| 25 | OR2T1 | na | 1986 | 0.357 | 0.0948 | Yes |
| 26 | OR5H2 | na | 2015 | 0.356 | 0.0989 | Yes |
| 27 | OR2D3 | na | 2278 | 0.336 | 0.0958 | Yes |
| 28 | OR5AS1 | na | 2310 | 0.334 | 0.0994 | Yes |
| 29 | OR1N2 | na | 2390 | 0.329 | 0.1016 | Yes |
| 30 | OR1A2 | na | 2408 | 0.328 | 0.1056 | Yes |
| 31 | OR8S1 | na | 2423 | 0.327 | 0.1096 | Yes |
| 32 | OR8B4 | na | 2460 | 0.326 | 0.1130 | Yes |
| 33 | OR52B6 | na | 2533 | 0.321 | 0.1152 | Yes |
| 34 | OR5M8 | na | 2691 | 0.312 | 0.1149 | Yes |
| 35 | OR4S1 | na | 2756 | 0.309 | 0.1172 | Yes |
| 36 | OR13C3 | na | 2777 | 0.307 | 0.1208 | Yes |
| 37 | OR10X1 | na | 2852 | 0.303 | 0.1228 | Yes |
| 38 | OR2AT4 | na | 3065 | 0.291 | 0.1206 | Yes |
| 39 | OR6V1 | na | 3071 | 0.291 | 0.1244 | Yes |
| 40 | OR10T2 | na | 3117 | 0.289 | 0.1270 | Yes |
| 41 | OR5F1 | na | 3177 | 0.287 | 0.1292 | Yes |
| 42 | OR52E6 | na | 3259 | 0.282 | 0.1306 | Yes |
| 43 | OR2A12 | na | 3486 | 0.272 | 0.1278 | Yes |
| 44 | OR6C76 | na | 3489 | 0.272 | 0.1314 | Yes |
| 45 | OR7C1 | na | 3498 | 0.272 | 0.1349 | Yes |
| 46 | OR10A6 | na | 3583 | 0.268 | 0.1361 | Yes |
| 47 | OR5B3 | na | 3631 | 0.266 | 0.1383 | Yes |
| 48 | OR8K1 | na | 3674 | 0.265 | 0.1407 | Yes |
| 49 | OR2G6 | na | 3803 | 0.261 | 0.1405 | Yes |
| 50 | OR2T2 | na | 3831 | 0.260 | 0.1432 | Yes |
| 51 | OR10AG1 | na | 3846 | 0.259 | 0.1463 | Yes |
| 52 | OR5D14 | na | 3865 | 0.259 | 0.1493 | Yes |
| 53 | OR2M4 | na | 3893 | 0.258 | 0.1521 | Yes |
| 54 | OR8D2 | na | 3896 | 0.258 | 0.1555 | Yes |
| 55 | OR3A1 | na | 3919 | 0.257 | 0.1583 | Yes |
| 56 | OR10G7 | na | 3935 | 0.257 | 0.1614 | Yes |
| 57 | OR10R2 | na | 3991 | 0.254 | 0.1632 | Yes |
| 58 | OR6B2 | na | 4099 | 0.251 | 0.1635 | Yes |
| 59 | OR6N1 | na | 4122 | 0.250 | 0.1663 | Yes |
| 60 | OR4S2 | na | 4128 | 0.250 | 0.1695 | Yes |
| 61 | OR6C2 | na | 4207 | 0.247 | 0.1706 | Yes |
| 62 | OR5AR1 | na | 4268 | 0.245 | 0.1722 | Yes |
| 63 | OR2T6 | na | 4279 | 0.245 | 0.1752 | Yes |
| 64 | CAMK2A | na | 4292 | 0.245 | 0.1782 | Yes |
| 65 | OR1F1 | na | 4328 | 0.243 | 0.1805 | Yes |
| 66 | OR1N1 | na | 4350 | 0.242 | 0.1832 | Yes |
| 67 | CNGA4 | na | 4362 | 0.242 | 0.1861 | Yes |
| 68 | OR2F1 | na | 4418 | 0.241 | 0.1878 | Yes |
| 69 | OR10S1 | na | 4435 | 0.240 | 0.1906 | Yes |
| 70 | OR6C65 | na | 4451 | 0.239 | 0.1934 | Yes |
| 71 | OR9K2 | na | 4462 | 0.239 | 0.1964 | Yes |
| 72 | OR13C9 | na | 4477 | 0.238 | 0.1992 | Yes |
| 73 | OR2T11 | na | 4510 | 0.237 | 0.2015 | Yes |
| 74 | OR56B4 | na | 4565 | 0.236 | 0.2031 | Yes |
| 75 | OR14C36 | na | 4580 | 0.235 | 0.2059 | Yes |
| 76 | OR10A2 | na | 4666 | 0.233 | 0.2066 | Yes |
| 77 | OR14A16 | na | 4725 | 0.231 | 0.2080 | Yes |
| 78 | OR5T1 | na | 4837 | 0.227 | 0.2079 | Yes |
| 79 | OR2A14 | na | 4900 | 0.225 | 0.2091 | Yes |
| 80 | OR10Q1 | na | 4931 | 0.225 | 0.2113 | Yes |
| 81 | OR10V1 | na | 4938 | 0.224 | 0.2142 | Yes |
| 82 | OR51F2 | na | 4988 | 0.223 | 0.2158 | Yes |
| 83 | OR6C1 | na | 5034 | 0.221 | 0.2175 | Yes |
| 84 | OR2C3 | na | 5248 | 0.215 | 0.2142 | Yes |
| 85 | OR5B17 | na | 5279 | 0.214 | 0.2162 | Yes |
| 86 | OR56A1 | na | 5342 | 0.212 | 0.2173 | Yes |
| 87 | OR3A2 | na | 5414 | 0.210 | 0.2181 | Yes |
| 88 | OR5M1 | na | 5434 | 0.209 | 0.2204 | Yes |
| 89 | CALML6 | na | 5567 | 0.206 | 0.2193 | Yes |
| 90 | OR4K13 | na | 5704 | 0.201 | 0.2181 | Yes |
| 91 | OR2L2 | na | 5804 | 0.199 | 0.2179 | Yes |
| 92 | OR6T1 | na | 5851 | 0.197 | 0.2192 | Yes |
| 93 | OR10A7 | na | 5864 | 0.197 | 0.2216 | Yes |
| 94 | OR5B21 | na | 5916 | 0.195 | 0.2227 | Yes |
| 95 | OR13G1 | na | 5953 | 0.194 | 0.2243 | Yes |
| 96 | OR4A16 | na | 6038 | 0.191 | 0.2245 | Yes |
| 97 | OR8B12 | na | 6110 | 0.189 | 0.2250 | Yes |
| 98 | OR8K5 | na | 6248 | 0.184 | 0.2235 | Yes |
| 99 | OR5D18 | na | 6429 | 0.180 | 0.2207 | Yes |
| 100 | OR2L3 | na | 6452 | 0.180 | 0.2225 | Yes |
| 101 | OR51Q1 | na | 6585 | 0.176 | 0.2210 | Yes |
| 102 | OR6C6 | na | 6639 | 0.174 | 0.2218 | Yes |
| 103 | OR2T12 | na | 6766 | 0.171 | 0.2205 | Yes |
| 104 | OR4P4 | na | 6786 | 0.170 | 0.2223 | Yes |
| 105 | OR5AN1 | na | 6862 | 0.168 | 0.2224 | Yes |
| 106 | OR52K1 | na | 6875 | 0.168 | 0.2243 | Yes |
| 107 | OR2T34 | na | 6927 | 0.166 | 0.2251 | Yes |
| 108 | OR1C1 | na | 6987 | 0.164 | 0.2256 | Yes |
| 109 | OR4C46 | na | 7001 | 0.164 | 0.2274 | Yes |
| 110 | OR2W3 | na | 7148 | 0.160 | 0.2253 | Yes |
| 111 | OR1L1 | na | 7155 | 0.160 | 0.2273 | Yes |
| 112 | OR2M7 | na | 7160 | 0.160 | 0.2294 | Yes |
| 113 | CALML5 | na | 7292 | 0.156 | 0.2277 | Yes |
| 114 | OR5K1 | na | 7306 | 0.156 | 0.2294 | Yes |
| 115 | OR5L2 | na | 7323 | 0.155 | 0.2311 | Yes |
| 116 | OR2A2 | na | 7405 | 0.153 | 0.2308 | Yes |
| 117 | OR13D1 | na | 7507 | 0.151 | 0.2299 | Yes |
| 118 | OR1B1 | na | 7586 | 0.149 | 0.2296 | Yes |
| 119 | OR5D16 | na | 7621 | 0.148 | 0.2306 | Yes |
| 120 | OR7A5 | na | 7634 | 0.148 | 0.2323 | Yes |
| 121 | OR6K6 | na | 7747 | 0.144 | 0.2310 | No |
| 122 | OR6K3 | na | 7820 | 0.143 | 0.2308 | No |
| 123 | OR6C75 | na | 7937 | 0.140 | 0.2294 | No |
| 124 | OR5T3 | na | 8204 | 0.133 | 0.2234 | No |
| 125 | OR10G8 | na | 8273 | 0.132 | 0.2233 | No |
| 126 | CNGB1 | na | 8349 | 0.130 | 0.2228 | No |
| 127 | OR6C74 | na | 8415 | 0.129 | 0.2227 | No |
| 128 | OR11G2 | na | 8604 | 0.125 | 0.2189 | No |
| 129 | OR10J1 | na | 8660 | 0.123 | 0.2190 | No |
| 130 | OR7D4 | na | 8868 | 0.119 | 0.2146 | No |
| 131 | OR9A4 | na | 8998 | 0.116 | 0.2124 | No |
| 132 | OR13C2 | na | 9319 | 0.109 | 0.2046 | No |
| 133 | OR9Q2 | na | 9360 | 0.108 | 0.2049 | No |
| 134 | OR10A5 | na | 9727 | 0.101 | 0.1956 | No |
| 135 | OR8A1 | na | 9978 | 0.096 | 0.1896 | No |
| 136 | OR4Q3 | na | 10272 | 0.091 | 0.1823 | No |
| 137 | OR2T10 | na | 10284 | 0.091 | 0.1833 | No |
| 138 | OR4C3 | na | 10474 | 0.087 | 0.1789 | No |
| 139 | OR5C1 | na | 10583 | 0.085 | 0.1769 | No |
| 140 | OR1J1 | na | 10811 | 0.081 | 0.1714 | No |
| 141 | OR1L4 | na | 10860 | 0.080 | 0.1711 | No |
| 142 | CAMK2G | na | 10986 | 0.078 | 0.1686 | No |
| 143 | OR2B11 | na | 11017 | 0.078 | 0.1687 | No |
| 144 | OR5P3 | na | 11153 | 0.076 | 0.1658 | No |
| 145 | OR6A2 | na | 11200 | 0.075 | 0.1655 | No |
| 146 | OR10Z1 | na | 11417 | 0.072 | 0.1602 | No |
| 147 | CLCA4 | na | 11467 | 0.071 | 0.1597 | No |
| 148 | OR51A4 | na | 11603 | 0.069 | 0.1568 | No |
| 149 | PRKX | na | 11663 | 0.068 | 0.1560 | No |
| 150 | OR11H4 | na | 11814 | 0.066 | 0.1525 | No |
| 151 | OR51B5 | na | 11898 | 0.065 | 0.1510 | No |
| 152 | OR5K2 | na | 11908 | 0.065 | 0.1516 | No |
| 153 | OR4C11 | na | 11915 | 0.065 | 0.1523 | No |
| 154 | OR51E2 | na | 11916 | 0.065 | 0.1532 | No |
| 155 | CALM2 | na | 11947 | 0.064 | 0.1532 | No |
| 156 | OR5M11 | na | 12119 | 0.062 | 0.1490 | No |
| 157 | OR1D2 | na | 12135 | 0.062 | 0.1494 | No |
| 158 | OR6F1 | na | 12710 | 0.054 | 0.1334 | No |
| 159 | OR6B1 | na | 12794 | 0.053 | 0.1317 | No |
| 160 | OR6K2 | na | 12827 | 0.052 | 0.1315 | No |
| 161 | OR6B3 | na | 12954 | 0.051 | 0.1285 | No |
| 162 | OR2S2 | na | 13069 | 0.049 | 0.1259 | No |
| 163 | OR52E4 | na | 13074 | 0.049 | 0.1264 | No |
| 164 | OR51L1 | na | 13281 | 0.046 | 0.1211 | No |
| 165 | OR51A7 | na | 13406 | 0.045 | 0.1181 | No |
| 166 | OR2Z1 | na | 13506 | 0.044 | 0.1158 | No |
| 167 | OR10P1 | na | 13540 | 0.043 | 0.1154 | No |
| 168 | OR52A1 | na | 13621 | 0.042 | 0.1137 | No |
| 169 | OR6S1 | na | 13649 | 0.042 | 0.1135 | No |
| 170 | OR4C6 | na | 13685 | 0.042 | 0.1130 | No |
| 171 | OR10H2 | na | 13831 | 0.040 | 0.1093 | No |
| 172 | OR51V1 | na | 13864 | 0.040 | 0.1089 | No |
| 173 | OR6M1 | na | 13904 | 0.039 | 0.1083 | No |
| 174 | OR5T2 | na | 14006 | 0.038 | 0.1059 | No |
| 175 | OR14I1 | na | 14447 | 0.033 | 0.0935 | No |
| 176 | OR10G2 | na | 14477 | 0.033 | 0.0931 | No |
| 177 | OR56B1 | na | 14670 | 0.031 | 0.0880 | No |
| 178 | OR4X1 | na | 14787 | 0.030 | 0.0850 | No |
| 179 | OR2V2 | na | 14943 | 0.028 | 0.0809 | No |
| 180 | OR4C12 | na | 14997 | 0.028 | 0.0797 | No |
| 181 | OR56A4 | na | 15043 | 0.027 | 0.0788 | No |
| 182 | OR9Q1 | na | 15107 | 0.027 | 0.0773 | No |
| 183 | OR4K5 | na | 15230 | 0.026 | 0.0741 | No |
| 184 | OR5M9 | na | 15285 | 0.025 | 0.0729 | No |
| 185 | OR8G1 | na | 15527 | 0.023 | 0.0662 | No |
| 186 | OR4K1 | na | 15561 | 0.022 | 0.0655 | No |
| 187 | OR52I2 | na | 15573 | 0.022 | 0.0655 | No |
| 188 | OR2AG2 | na | 15675 | 0.021 | 0.0628 | No |
| 189 | OR4N5 | na | 15987 | 0.018 | 0.0540 | No |
| 190 | OR2AK2 | na | 16230 | 0.015 | 0.0472 | No |
| 191 | OR10G9 | na | 16283 | 0.015 | 0.0459 | No |
| 192 | OR10K2 | na | 16418 | 0.014 | 0.0422 | No |
| 193 | OR8D4 | na | 16837 | 0.010 | 0.0301 | No |
| 194 | OR4K2 | na | 16839 | 0.010 | 0.0302 | No |
| 195 | GUCY2D | na | 16847 | 0.010 | 0.0302 | No |
| 196 | OR51T1 | na | 16963 | 0.009 | 0.0269 | No |
| 197 | CLCA1 | na | 17121 | 0.008 | 0.0225 | No |
| 198 | CALML3 | na | 17129 | 0.008 | 0.0224 | No |
| 199 | ARRB2 | na | 17178 | 0.007 | 0.0211 | No |
| 200 | CALM1 | na | 17693 | 0.002 | 0.0061 | No |
| 201 | OR2G3 | na | 17825 | 0.001 | 0.0023 | No |
| 202 | OR52N2 | na | 18006 | -0.001 | -0.0029 | No |
| 203 | OR8I2 | na | 18094 | -0.001 | -0.0054 | No |
| 204 | OR52J3 | na | 18177 | -0.002 | -0.0078 | No |
| 205 | OR52D1 | na | 18230 | -0.003 | -0.0092 | No |
| 206 | OR1Q1 | na | 18306 | -0.004 | -0.0114 | No |
| 207 | OR2B3 | na | 18333 | -0.004 | -0.0121 | No |
| 208 | OR4B1 | na | 18462 | -0.005 | -0.0157 | No |
| 209 | OR5AK2 | na | 18481 | -0.005 | -0.0162 | No |
| 210 | OR3A3 | na | 18639 | -0.007 | -0.0207 | No |
| 211 | OR56A3 | na | 18674 | -0.007 | -0.0216 | No |
| 212 | OR10H5 | na | 18728 | -0.007 | -0.0230 | No |
| 213 | OR1S1 | na | 19312 | -0.014 | -0.0398 | No |
| 214 | OR1L6 | na | 19401 | -0.015 | -0.0421 | No |
| 215 | PRKACA | na | 19584 | -0.017 | -0.0472 | No |
| 216 | OR2AG1 | na | 19855 | -0.020 | -0.0548 | No |
| 217 | OR1K1 | na | 20115 | -0.023 | -0.0620 | No |
| 218 | OR51F1 | na | 20358 | -0.026 | -0.0687 | No |
| 219 | OR2D2 | na | 20409 | -0.027 | -0.0698 | No |
| 220 | OR5J2 | na | 20530 | -0.028 | -0.0729 | No |
| 221 | OR4C16 | na | 20596 | -0.029 | -0.0744 | No |
| 222 | OR4D1 | na | 20671 | -0.030 | -0.0762 | No |
| 223 | OR2C1 | na | 20737 | -0.030 | -0.0777 | No |
| 224 | GNAL | na | 20832 | -0.031 | -0.0800 | No |
| 225 | CLCA2 | na | 21115 | -0.035 | -0.0877 | No |
| 226 | OR7E24 | na | 21194 | -0.036 | -0.0895 | No |
| 227 | OR14J1 | na | 21269 | -0.037 | -0.0911 | No |
| 228 | OR5V1 | na | 21691 | -0.042 | -0.1028 | No |
| 229 | OR2AE1 | na | 21704 | -0.042 | -0.1026 | No |
| 230 | OR9G4 | na | 21848 | -0.044 | -0.1062 | No |
| 231 | OR4D6 | na | 22085 | -0.047 | -0.1124 | No |
| 232 | OR6C4 | na | 22185 | -0.048 | -0.1146 | No |
| 233 | OR52K2 | na | 22501 | -0.052 | -0.1231 | No |
| 234 | OR4A47 | na | 22650 | -0.054 | -0.1267 | No |
| 235 | OR2H1 | na | 22855 | -0.057 | -0.1318 | No |
| 236 | OR1J4 | na | 23109 | -0.061 | -0.1384 | No |
| 237 | OR51B6 | na | 23124 | -0.061 | -0.1379 | No |
| 238 | OR4X2 | na | 23270 | -0.064 | -0.1413 | No |
| 239 | OR6Y1 | na | 23275 | -0.064 | -0.1405 | No |
| 240 | OR2M5 | na | 23317 | -0.064 | -0.1408 | No |
| 241 | OR2M2 | na | 23461 | -0.067 | -0.1441 | No |
| 242 | OR7A17 | na | 23462 | -0.067 | -0.1432 | No |
| 243 | OR4N2 | na | 23475 | -0.067 | -0.1426 | No |
| 244 | PRKG2 | na | 23519 | -0.068 | -0.1430 | No |
| 245 | CALM3 | na | 23602 | -0.069 | -0.1444 | No |
| 246 | OR6Q1 | na | 23616 | -0.070 | -0.1438 | No |
| 247 | OR5L1 | na | 23624 | -0.070 | -0.1431 | No |
| 248 | OR13H1 | na | 23722 | -0.071 | -0.1450 | No |
| 249 | OR5A2 | na | 24045 | -0.077 | -0.1533 | No |
| 250 | OR8G5 | na | 24758 | -0.089 | -0.1728 | No |
| 251 | OR51G1 | na | 24891 | -0.091 | -0.1754 | No |
| 252 | OR1I1 | na | 24942 | -0.092 | -0.1756 | No |
| 253 | OR4A15 | na | 25137 | -0.096 | -0.1800 | No |
| 254 | OR1A1 | na | 25159 | -0.096 | -0.1793 | No |
| 255 | OR7G1 | na | 25365 | -0.100 | -0.1839 | No |
| 256 | OR4F15 | na | 25706 | -0.107 | -0.1923 | No |
| 257 | OR13J1 | na | 25806 | -0.109 | -0.1937 | No |
| 258 | OR2T33 | na | 25998 | -0.113 | -0.1978 | No |
| 259 | OR2T8 | na | 26144 | -0.116 | -0.2004 | No |
| 260 | OR10C1 | na | 26201 | -0.117 | -0.2004 | No |
| 261 | OR2W1 | na | 26393 | -0.121 | -0.2044 | No |
| 262 | OR4K14 | na | 26409 | -0.122 | -0.2031 | No |
| 263 | OR2L8 | na | 26540 | -0.124 | -0.2052 | No |
| 264 | OR4C15 | na | 26558 | -0.124 | -0.2041 | No |
| 265 | OR11A1 | na | 26564 | -0.125 | -0.2025 | No |
| 266 | OR51B4 | na | 26627 | -0.126 | -0.2026 | No |
| 267 | OR12D2 | na | 26654 | -0.127 | -0.2016 | No |
| 268 | OR4D2 | na | 26799 | -0.130 | -0.2041 | No |
| 269 | OR10J3 | na | 26857 | -0.132 | -0.2039 | No |
| 270 | ADCY3 | na | 26955 | -0.134 | -0.2049 | No |
| 271 | PDC | na | 27007 | -0.135 | -0.2046 | No |
| 272 | OR2T4 | na | 27054 | -0.136 | -0.2041 | No |
| 273 | OR10G4 | na | 27091 | -0.137 | -0.2032 | No |
| 274 | GUCA1A | na | 27120 | -0.138 | -0.2022 | No |
| 275 | OR5M10 | na | 27149 | -0.139 | -0.2011 | No |
| 276 | OR6C68 | na | 27252 | -0.141 | -0.2022 | No |
| 277 | OR52N5 | na | 27454 | -0.146 | -0.2060 | No |
| 278 | OR4D11 | na | 27519 | -0.148 | -0.2059 | No |
| 279 | OR5AU1 | na | 27735 | -0.154 | -0.2100 | No |
| 280 | OR4D10 | na | 27769 | -0.155 | -0.2089 | No |
| 281 | OR13C4 | na | 28097 | -0.163 | -0.2162 | No |
| 282 | OR7D2 | na | 28103 | -0.163 | -0.2141 | No |
| 283 | OR9G1 | na | 28105 | -0.164 | -0.2119 | No |
| 284 | GUCA1B | na | 28130 | -0.164 | -0.2104 | No |
| 285 | OR10K1 | na | 28721 | -0.182 | -0.2251 | No |
| 286 | OR4D5 | na | 28732 | -0.183 | -0.2229 | No |
| 287 | OR11L1 | na | 28898 | -0.188 | -0.2252 | No |
| 288 | OR2L13 | na | 28940 | -0.189 | -0.2238 | No |
| 289 | OR52A5 | na | 28993 | -0.191 | -0.2227 | No |
| 290 | PDE1C | na | 29203 | -0.197 | -0.2261 | No |
| 291 | OR12D3 | na | 29309 | -0.200 | -0.2265 | No |
| 292 | OR13C8 | na | 29344 | -0.201 | -0.2247 | No |
| 293 | OR52M1 | na | 29393 | -0.203 | -0.2234 | No |
| 294 | OR5D13 | na | 29397 | -0.203 | -0.2207 | No |
| 295 | OR51B2 | na | 29458 | -0.204 | -0.2197 | No |
| 296 | OR4D9 | na | 29539 | -0.207 | -0.2192 | No |
| 297 | OR7G3 | na | 29654 | -0.212 | -0.2197 | No |
| 298 | OR8B8 | na | 29669 | -0.212 | -0.2172 | No |
| 299 | OR4E2 | na | 29707 | -0.213 | -0.2154 | No |
| 300 | OR5B12 | na | 29718 | -0.214 | -0.2127 | No |
| 301 | OR51A2 | na | 29720 | -0.214 | -0.2099 | No |
| 302 | OR2M3 | na | 29734 | -0.215 | -0.2073 | No |
| 303 | OR4C45 | na | 29752 | -0.215 | -0.2049 | No |
| 304 | OR2K2 | na | 29790 | -0.217 | -0.2030 | No |
| 305 | OR52L1 | na | 29894 | -0.220 | -0.2031 | No |
| 306 | OR7G2 | na | 29996 | -0.223 | -0.2030 | No |
| 307 | OR52N1 | na | 30027 | -0.224 | -0.2008 | No |
| 308 | CNGA3 | na | 30040 | -0.225 | -0.1981 | No |
| 309 | OR51G2 | na | 30075 | -0.226 | -0.1960 | No |
| 310 | OR6C3 | na | 30082 | -0.226 | -0.1931 | No |
| 311 | OR52B4 | na | 30177 | -0.230 | -0.1927 | No |
| 312 | OR5H6 | na | 30368 | -0.237 | -0.1950 | No |
| 313 | OR5AP2 | na | 30395 | -0.238 | -0.1926 | No |
| 314 | OR2F2 | na | 30492 | -0.242 | -0.1921 | No |
| 315 | OR2T27 | na | 30655 | -0.249 | -0.1934 | No |
| 316 | OR1M1 | na | 30671 | -0.249 | -0.1905 | No |
| 317 | OR2J3 | na | 30682 | -0.249 | -0.1874 | No |
| 318 | OR6C70 | na | 30719 | -0.251 | -0.1850 | No |
| 319 | OR5AC2 | na | 30816 | -0.255 | -0.1843 | No |
| 320 | OR5A1 | na | 30818 | -0.255 | -0.1809 | No |
| 321 | PRKG1 | na | 30895 | -0.258 | -0.1796 | No |
| 322 | OR2B2 | na | 30963 | -0.262 | -0.1780 | No |
| 323 | OR4L1 | na | 30996 | -0.263 | -0.1754 | No |
| 324 | OR5M3 | na | 31087 | -0.268 | -0.1744 | No |
| 325 | OR2T5 | na | 31353 | -0.283 | -0.1782 | No |
| 326 | PRKACG | na | 31385 | -0.285 | -0.1753 | No |
| 327 | OR2A42 | na | 31413 | -0.286 | -0.1722 | No |
| 328 | OR2A5 | na | 31440 | -0.288 | -0.1690 | No |
| 329 | OR5I1 | na | 31467 | -0.289 | -0.1658 | No |
| 330 | OR7A10 | na | 31469 | -0.289 | -0.1619 | No |
| 331 | OR8J3 | na | 31526 | -0.292 | -0.1596 | No |
| 332 | OR1L3 | na | 31538 | -0.293 | -0.1560 | No |
| 333 | OR51I1 | na | 31590 | -0.295 | -0.1534 | No |
| 334 | OR13C5 | na | 31644 | -0.298 | -0.1509 | No |
| 335 | OR51I2 | na | 31837 | -0.309 | -0.1523 | No |
| 336 | CAMK2D | na | 31910 | -0.312 | -0.1502 | No |
| 337 | OR51S1 | na | 31945 | -0.314 | -0.1469 | No |
| 338 | OR2B6 | na | 31976 | -0.316 | -0.1435 | No |
| 339 | OR10H1 | na | 32067 | -0.321 | -0.1418 | No |
| 340 | OR9A2 | na | 32188 | -0.329 | -0.1408 | No |
| 341 | OR8K3 | na | 32235 | -0.332 | -0.1376 | No |
| 342 | OR7C2 | na | 32401 | -0.343 | -0.1378 | No |
| 343 | OR52N4 | na | 32515 | -0.352 | -0.1363 | No |
| 344 | OR52I1 | na | 32556 | -0.355 | -0.1326 | No |
| 345 | OR4A5 | na | 32599 | -0.357 | -0.1290 | No |
| 346 | OR8H3 | na | 32720 | -0.366 | -0.1275 | No |
| 347 | OR52E2 | na | 32774 | -0.370 | -0.1240 | No |
| 348 | OR1G1 | na | 33148 | -0.402 | -0.1294 | No |
| 349 | OR2H2 | na | 33184 | -0.405 | -0.1250 | No |
| 350 | OR4M1 | na | 33213 | -0.409 | -0.1202 | No |
| 351 | OR8U8 | na | 33316 | -0.421 | -0.1175 | No |
| 352 | OR2Y1 | na | 33400 | -0.431 | -0.1140 | No |
| 353 | OR2J2 | na | 33416 | -0.432 | -0.1086 | No |
| 354 | PRKACB | na | 33438 | -0.435 | -0.1033 | No |
| 355 | OR13A1 | na | 33647 | -0.462 | -0.1031 | No |
| 356 | OR51D1 | na | 33659 | -0.463 | -0.0971 | No |
| 357 | OR1E1 | na | 33711 | -0.472 | -0.0922 | No |
| 358 | OR8H2 | na | 33719 | -0.473 | -0.0860 | No |
| 359 | OR11H6 | na | 33761 | -0.481 | -0.0807 | No |
| 360 | OR1S2 | na | 33803 | -0.486 | -0.0752 | No |
| 361 | OR51M1 | na | 33839 | -0.494 | -0.0696 | No |
| 362 | OR8U1 | na | 33857 | -0.497 | -0.0633 | No |
| 363 | OR52W1 | na | 33912 | -0.506 | -0.0580 | No |
| 364 | OR10H4 | na | 33985 | -0.519 | -0.0531 | No |
| 365 | GUCA1C | na | 34131 | -0.551 | -0.0498 | No |
| 366 | OR4C13 | na | 34145 | -0.556 | -0.0426 | No |
| 367 | OR1J2 | na | 34284 | -0.596 | -0.0385 | No |
| 368 | OR2A7 | na | 34385 | -0.627 | -0.0329 | No |
| 369 | OR8H1 | na | 34430 | -0.645 | -0.0255 | No |
| 370 | OR2A25 | na | 34511 | -0.683 | -0.0185 | No |
| 371 | OR10J5 | na | 34578 | -0.724 | -0.0106 | No |
| 372 | OR52R1 | na | 34718 | -1.102 | 0.0003 | No |
Table: GSEA details [plain text format]

  

Fig 2: KEGG\_OLFACTORY\_TRANSDUCTION      
 Blue-Pink O' Gram in the Space of the Analyzed GeneSet

  

Fig 3: KEGG\_OLFACTORY\_TRANSDUCTION: Random ES distribution      
 Gene set null distribution of ES for **KEGG\_OLFACTORY\_TRANSDUCTION**
